# When altruists cannot help: the influence of altruism on the mental health of university students during the COVID-19 pandemic

**DOI:** 10.1186/s12992-020-00587-y

**Published:** 2020-07-10

**Authors:** Yi Feng, Min Zong, Zhizun Yang, Wen Gu, Dan Dong, Zhihong Qiao

**Affiliations:** 1 School of Psychology, Beijing Normal University, No.19, Xinjiekouwai St, Haidian District, Beijing, 100875 China; 2 Mental Health Center, Central University of Finance and Economics, Beijing, China; 3 Mental Health Center, China Foreign Affairs University, Beijing, China

**Keywords:** Altruism, Mental health, Negative affect, Perceived risk, COVID-19

## Abstract

**Background:**

The positive predictive effect of altruism on physical and psychological well-being has been extensively demonstrated in previous studies, but few studies have examined the effect of altruism on negative mental health outcomes when altruists cannot perform altruistic behaviours. This study explored the influence of altruism on negative affect and mental health (anxiety and depressive symptoms) during the COVID-19 pandemic while people self-isolated at home in China.

**Method:**

University students were recruited to participate in a cross-sectional online survey during the outbreak of COVID-19 in China. Self-reported perceived risk, altruism, negative affect, anxiety and depressive symptoms were measured using the Self-Report Altruism Scale (SRA scale), the Positive and Negative Affect Schedule (PANAS), the 7-item Generalized Anxiety Disorder Scale (GAD-7) and the 9-item Patient Health Questionnaire depression scale (PHQ-9). A structural equation model was used to analyse the mediating and moderating effects on mental health.

**Results:**

The final sample comprised 1346 Chinese participants (Mage = 19.76 ± 2.23 years, 73% female). Overall, the higher the risk the participants perceived, the more negative affect they exhibited (*β* = 0.16, *p* < .001), and thus, the more anxious and depressed they felt (*β* = 0.134, *p* < .001); however, this relationship between risk perception and negative affect was moderated by altruism. In contrast to previous studies, the increase in negative affect associated with the increased perceived risk was pronounced among individuals with high altruism (*t* = 7.68, *p* < .001).

**Conclusions:**

Individuals with high altruism exhibited more negative affect than those with low altruism, which indirectly increased their anxiety and depressive symptoms. These findings enrich theories of altruism and provide valuable insight into the influence of altruism on mental health during the COVID-19 outbreak.

## Introduction

Coined as a “once-in-a-century pandemic”, COVID-19 has been an ongoing global emergency due to its high mortality rate and widespread contagion [1]. In the face of the health threat of COVID-19, people have shown predictable threat responses, including fear, anxiety, depression, panic shopping, and xenophobic tendencies [2]. However, not everyone responds in the same way. Many psychological factors shape people’s threat reactions, such as risk perception, personality traits, and social support [3].

### Risk perception and mental health

Stress-appraisal theory (Lazarus and Folkman, 1984) posits that the perceived threat of a health risk depends on the perceived vulnerability to risk and the ability to cope with it [4]. COVID-19 has evoked a widespread and worldwide threat to health due to vulnerability to the virus or difficulty coping with it. Research concerning risk perception and mental health in cases of emerging infectious disease is still relatively limited. Although perceived risk is not the same as actual risk in most cases, perceived risk still leads to negative emotions and mental health outcomes, such as anxiety and depression [5, 6]. It is urgent to investigate the influence of risk perception on negative emotion and public mental health during the COVID-19 pandemic.

### Altruism and mental health

As an important concept in social psychology, altruism is highly valued for its profound importance to human evolution and the development of human civilization [7]. Altruism was first defined by Comte as “an unselfish regard for the welfare of others” [8], and it comprises the following two core concepts: empathy (altruistic attitude) and prosocial behaviour (altruistic behaviour). Currently, the dominant perspective for understanding altruism suggests that altruists receive instant or long-term physical and psychological benefits from engaging in altruistic activities [9]. Altruistic behaviour is associated with reduced aggression, better physical and mental health, longevity, and improved well-being [10–12]. Similarly, altruistic behaviours are positively correlated with happiness, responsibility and social adaptation among university students [13, 14]. Three models have been proposed to explain the connection between altruism and health. The evolutionary biology model suggests that altruistic behaviour within groups confers a competitive advantage against other groups. The physiological advantages model claims that altruistic emotions gain dominance over anxiety and fear and reduce the stress caused by the fight-fight response in the face of perceived danger. The positive emotion model explains that the positive emotions (such as kindness, compassion, and other-regarding love) induced by altruism enhance health by displacing negative emotions [11]. The incentive factors of altruism, such as enhanced self-efficacy as an agentic mechanism and increased social worth as a communal mechanism, also provide possible explanations for engagement in altruistic activities [15]. Empathy is widely considered an essential premise for altruistic behaviour [16]. Nonetheless, few studies have explored the psychological outcomes of altruists with empathy when altruistic behaviours cannot be performed under specific circumstances. The outbreak of COVID-19 represents a context in which most people in China self-isolated at home under the policy of social distancing. Most altruists could only remain at home and access COVID-19-related information on social media instead of performing direct altruistic behaviours during the pandemic. A neglected possibility is that altruism may not serve as a protective mental health factor against the threat of COVID-19 during the self-isolation period. We predict that compared to individuals with low altruism, those with high altruism may feel more anxious due to their empathy towards infected patients and more depressed due to their helplessness towards others. However, to the best of our knowledge, previous research has provided no guidance regarding altruists’ psychological responses in such a situation.

### Mechanism underlying the effect of altruism on mental health

There is no consensus among researchers regarding the mechanisms underlying the association between altruism and mental health. Theoretical works have suggested that altruism affects mental health through increased positive mood or decreased negative mood. Some studies have proposed that altruism accelerates elevated positive mood [17, 18], whereas other studies have argued that altruism buffers against negative mood [19]. The outbreak of COVID-19 provides a natural research context to explore this controversy. As noted above, we expect that people with high altruism may have had more negative mental health outcomes (anxiety and depressive symptoms) during the self-isolation period, which could verify the mediating role of positive or negative affect.

### Aims of this study

This article focuses on health psychology to formulate a theoretical model of psychological responses to the COVID-19 threat. First, we examine the effect of individual perceived risk on public mental health outcomes, including anxiety and depressive symptoms. Second, we attempt to extend the understanding of altruistic norms from a different perspective by examining the effect of altruism on emotional outcomes while focusing on conditions under which altruistic behaviour cannot occur. Finally, we clarify the mechanism underlying the effect of altruism on mental health by examining the mediating effect of positive and negative emotion on mental health. In brief, by combining the above corollaries, we hypothesize the following: (1) perceived risk will directly predict mental health (including anxiety and depressive symptoms); (2) perceived risk will predict negative affect, and the effect of perception on negative affect will be moderated by altruism; and (3) negative affect will mediate the effect of perceived risk on mental health (including anxiety and depressive symptoms).

## Methods

### Participants and sampling

This study adopted a cross-sectional design and was conducted during the outbreak of COVID-19 (February 8–28, 2020). Simple cluster sampling was used to construct the sample. University students from a Beijing university were recruited to complete an online questionnaire while they self-isolated at home under the government policy of social distancing and school closure. The students were from cities and towns located in different areas of China and were not only from Beijing; thus, the results yielded from this sample may be generalizable to a larger population of students in different areas throughout the country during this specific period. Participants who met the following criteria were included: (1) Chinese students; (2) students who were able to understand Chinese; and (3) students who were not diagnosed with COVID-19 and whose family members had not been diagnosed.

### Ethical approval and consent

The participants were informed about the purpose and procedures of this study via an online WeChat notification before the investigation. Online informed written consent was obtained from all participants at the beginning of the questionnaire. The research protocol was approved by the Research Ethics Review Committee of Beijing Normal University, China.

### Measures

The participants clicked a box to indicate their consent to complete an online survey concerning “University students’ psychological responses to COVID-19”. The survey consisted of a series of scales and demographic information. The demographic measures included age, sex, ethnic group, and location.

#### Perceived risk of COVID-19

Individual perceived risk was assessed by three items. Two items were related to perceived vulnerability to COVID-19 (i.e., “How likely do you think it is that you will be infected with COVID-19?” and “How likely do you think it is that your family members/relatives/friends will be infected with COVID-19?”) and were rated on an 11-point scale from 0 to 100%. The other item was related to the ability to cope with the pandemic: “How long do you think it will take to go back to normal life in your area?” The participants were given five choices (1 = within 1 month; 2 = 2–3 months; 3 = 4–6 months; 4 = 7–12 months; 5 = more than 1 year). The responses to these items formed the composite score of perceived risk (α = .70), with higher scores indicating a greater perceived risk.

#### Altruism

The Self-Report Altruism Scale (SRA scale) was adapted from the scale designed by Rushton, Chrisjohn and Fekken, and it includes 20 items concerning altruistic behaviours (e.g., “I have helped push a stranger’s car out of the snow” and “I have helped an acquaintance to move households”) [20]. The Chinese version of the SRA scale (C-SRA scale) has been validated (Cronbach’s α = .86) [21]. The participants clicked the option that best conformed to their past acts on a 5-point scale (1 = never; 2 = once, 3 = more than once; 4 = often; 5 = very often). We calculated a composite altruism composite score (α = .89), with higher scores indicating higher altruism.

#### Positive and negative affect

The Positive and Negative Affect Scale (PANAS) consisting of 20 items was used to measure emotional outcomes [22]. The PANAS comprises two scales, i.e., a 10-item positive affect scale (e.g., enthusiastic, excited, and inspired) and a 10-item negative affect scale (e.g., afraid, upset, and distressed). Previous studies have regarded positive and negative affect as two dominant and relatively independent dimensions rather than two poles on the same continuous scale [22, 23]. For each item, the participants were asked to indicate the extent to which they had felt the corresponding emotion in the past 2 weeks on a 5-point scale (1 = very slightly or not at all; 2 = a little, 3 = moderately; 4 = quite a bit; 5 = extremely). Composite positive (α = .92) and negative (α = .94) affect scores were calculated separately, with higher scores indicating higher positive or negative emotions.

#### Mental health

Anxiety and depression are the two most common indicators used to assess mental health in the general population and clinical practice [24, 25]. Anxiety and depression diagnoses frequently tend to co-occur, and their symptoms are highly correlated [26]. Thus, the latent variable “mental health” was constructed as an outcome variable based on anxiety and depressive symptoms in this study. The 7-item Generalized Anxiety Disorder Scale (GAD-7), which is a self-report screening scale, was used to assess anxiety symptoms [27]. The Chinese version of the GAD-7 has been validated and shown great reliability (Cronbach’s α = 0.89) [28]. The participants were asked to indicate the frequency of the occurrence of anxiety symptoms over the past 2 weeks on a 4-point Likert scale (0 = not at all; 1 = several days, 2 = more than half the days; 3 = nearly every day). A composite anxiety score was created (α = .92), with higher scores indicating anxiety symptoms of greater severity. Anxiety symptoms were identified based on a cut-off score of 5 in this study [27]. Similar to anxiety symptoms, depressive symptoms were measured through the self-report 9-item Patient Health Questionnaire (PHQ-9) [29], which has been verified in China (Cronbach’s α = 0.86) [30]. Each item assesses the frequency of the occurrence of depressive symptoms over the past 2 weeks on a 4-point scale from 0 (not at all) to 3 (nearly every day). We computed the composite depression score (α = .89). Higher scores indicated more severe depressive symptoms. The cut-off score for depressive symptoms was 5 in our study [29].

### Data analysis

We used the Shapiro-Wilk test to examine the normality of the continuous variables. We found that all continuous variables were not normally distributed. Comparisons of the demographic characteristics between the participants with and without anxiety symptoms and between those with and without depressive symptoms were conducted. A Mann-Whitney U test was performed for the age comparisons, and a chi-square test was performed for the sex and ethnic comparisons. Spearman correlations between the continuous variables measured above were calculated due to their non-normal distributions All above analyses were performed using IBM SPSS Statistics software version 23.0. In addition, the hypothesized moderating and mediating effects were examined by structural equation modelling (SEM) using Mplus software version 8.0. The goodness of fit was assessed by computing the comparative fit index (CFI), Tucker-Lewis index (TLI), root mean square error of approximation (RMSEA) and standardized root mean residual (SRMR) [31]. The acceptable levels of the goodness-of-fit model parameters are CFI > .90, TFI > .90, RMSEA < 08, and SRMR < .08 [32]. Moreover, to test the statistical significance of the moderating and mediating effects, we conducted bias-corrected bootstrap tests with 95% confidence intervals. The significance value was set at .05 in this study.

## Results

### Background characteristics and covariates

In total, 1493 questionnaires were distributed, and 1346 participants completed the survey. The response rate was 90.15%. The final sample comprised 1346 participants with the following characteristics: mean age = 19.76 ± 2.23 years; 364 (27.0%) males (Mage = 19.69 ± 2.30 years) and 982 (73.0%) females (Mage = 19.79 ± 2.21 years); 1143 (84.9%) of Han nationality (Mage = 19.81 ± 2.32 years) and 203 (15.1%) belonging to other ethnic groups (Mage = 19.48 ± 1.66 years). Table 1 shows the summary statistics of the participants’ background characteristics.

**Table 1:** Demographic characteristics of the study sample (*n* = 1346)

	Without anxiety symptoms (***N*** = 992)	With anxiety symptoms (***N*** = 354)	***p*** value
	*n* (%)	*n* (%)	
**Sex**			0.14
Male (*n* = 364)	279 (28.1%)	85 (24.0%)	
Female (*n* = 982)	713 (71.9%)	269 (76.0%)	
**Ethnic group**			0.78
Han (*n* = 1143)	844 (85.1%)	299 (84.5%)	
Others (*n* = 203)	148 (14.9%)	55 (15.5%)	
***Mean Age (SD)***	19.63 (2.15)	20.13 (2.40)	**< 0.001**
	**Without depression** **symptoms (*N*** **= 917)**	**With depression** **symptoms (*N*** **= 429)**	***p*value**
	***n (%)***	***n (%)***	
**Sex**			0.90
Male (*n* = 364)	247 (26.9%)	117 (27.3%)	
Female (*n* = 982)	670 (73.2%)	312 (72.7%)	
**Ethnic group**			0.39
Han (*n* = 1143)	784 (85.5%)	359 (83.7%)	
Others (*n* = 203)	133 (14.5%)	70 (16.3%)	
***Mean Age*(*SD*)**	19.63 (2.13)	20.04 (2.41)	**0.001**

*Note.* The cut-off score for with and without anxiety / depressive symptoms is 5 in this study

*p* value: Chi-square test for sex and ethnic comparisons; Mann-Whitney U test for age comparisons

### Correlations between mental health and other variables

Table 2 shows the results of the Spearman correlations between perceived risk, altruism, positive affect, negative affect, anxiety and depressive symptoms. Significant correlations were found between anxiety symptoms and perceived risk (*r* = 0.17, *p* < .01), positive affect (*r* = − 0.27, *p* < .01) and negative affect (*r* = 0.57, *p* < .01); depressive symptoms were significantly correlated with perceived risk (*r* = 0.18, *p* < .01), positive affect (*r* = − 0.42, *p* < .01), negative affect (*r* = 0.54, *p* < .01) and anxiety symptoms (*r* = 0.67, *p* < .01).

**Table 2:** Spearman’s correlation coefficients between main variables and covariates

	1	2	3	4	5	6	7	8
1 Sex	1							
2 Age	0.03	1						
3 Ethnicity	0.00	−0.03	1					
4 Perceived risk	0.08**	−0.11**	0.01	1				
5 Altruism	−0.07**	0.11**	0.02	−0.02	1			
6 Negative affect	0.09*	0.15**	0.06*	0.16**	0.05	1		
7 Positive affect	−0.06*	−0.08**	− 0.03	−0.13**	0.28**	−0.24**	1	
8 Anxiety	0.12*	0.13**	0.00	0.17**	0.02	0.57**	−0.27**	1
9 Depression	0.03	0.07*	0.03	0.18**	−0.05	0.54**	−0.42**	0.67**

*Note.* The sex variable was coded as “1 = male, 2 = female”

*p < .05, **p < .01

### Moderating effect of altruism

We conducted an SEM and path analysis to test our moderation hypothesis with perceived risk as a predictor and altruism as a moderator. Sex, age and ethnicity were controlled as covariates in the SEM. We separately examined the moderating effect of altruism on positive affect, negative affect and the mental health latent variable.

**Fig. 1:**
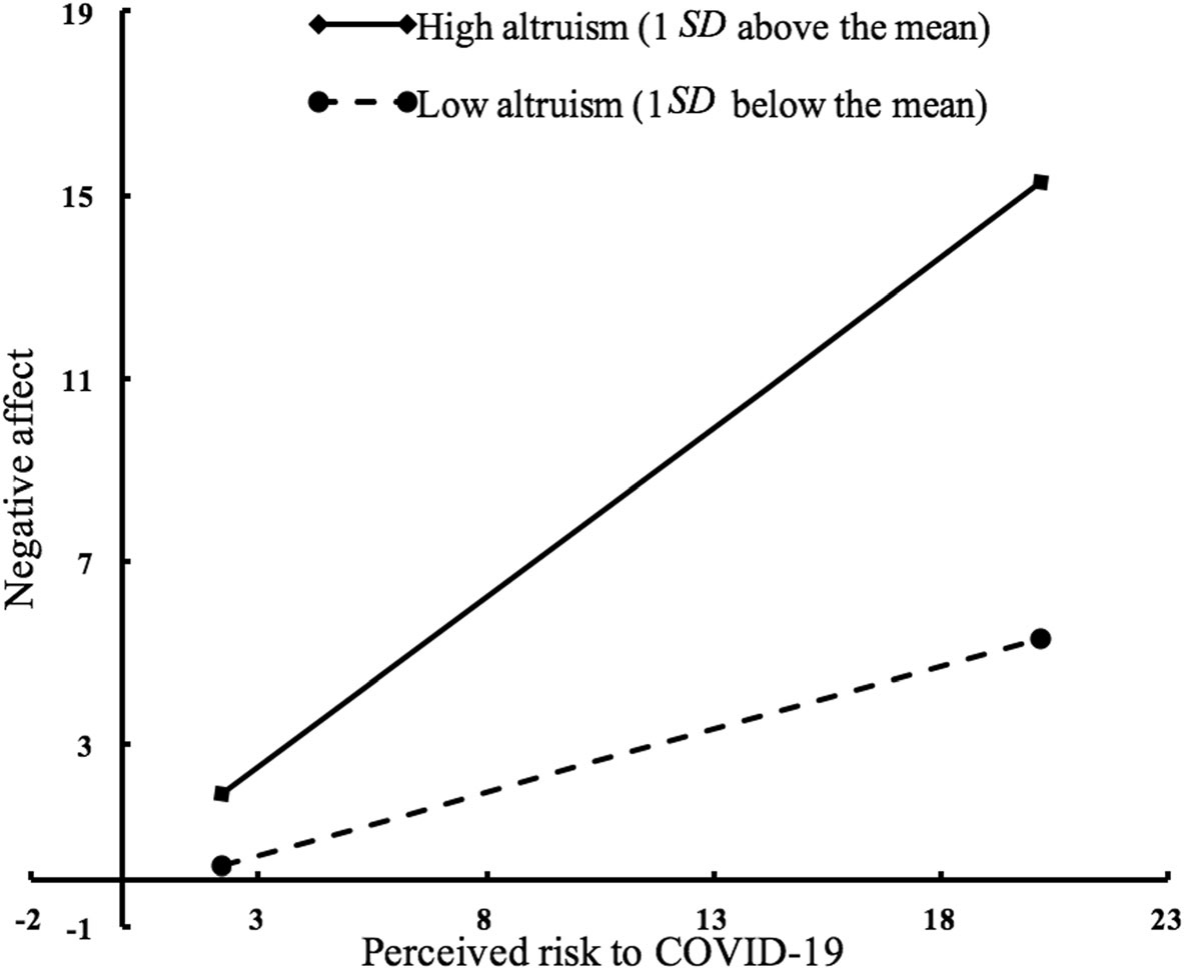
Interaction effect between perceived risk of COVID-19 and altruism on negative affect

First, we found that there was no interaction effect between altruism and perceived risk on positive affect (*β* = 0.05, 95% *CI* = [− 0.11, 0.11], *p* > .05). Second, there was no main effect of altruism on negative affect (*β* = 0.02, 95% *CI* = [− 0.02, 0.07], *p* > .05). We found a main effect of perceived risk on negative affect (*β* = 0.17, 95% *CI* = [0.12, 0.21], *p* < .001). However, altruism moderated this main effect, as indicated by a significant interaction effect between altruism and perceived risk on negative affect (*β* = 0.10, 95% *CI* = [0.03, 0.16], *p* < .05). Figure 1 shows the results of the moderating effect of altruism on negative affect. As illustrated in Fig. 1, at high levels of altruism (1 *SD* above the mean), greater perceived risk predicted greater negative affect (*t* = 7.68, 95% *CI* = [0.55, 0.94], *p* < .001). However, at low levels of altruism (1 *SD* below the mean), the relationship between perceived risk and negative affect was attenuated (*t* = 2.41, 95% *CI* = [0.05, 0.50], *p* < .001). In addition, the moderating role of altruism on negative affect was mainly driven by individuals who perceived a high risk of COVID-19. Among those who perceived a low risk of COVID-19, those with high altruism demonstrated the same level of negative affect (*M* = − 2.16, *SD* = 6.70) as those with low altruism (*M* = −.99, *SD* = 6.88, *t* = − 0.934, *p* > .05). However, among those who perceived a high risk of COVID-19, those with high altruism exhibited significantly greater negative affect (*M* = 5.80, *SD* = 9.36) than those with low altruism (*M* = − 2.09, *SD* = 5.85, *t* = 3.2, *p* < .01). Finally, for the latent mental health variable, the main effect of perceived risk on mental health was significant (*β* = 0.25, 95% *CI* = [0.17, 0.32], *p* < .001). Nevertheless, we found no main effect of altruism on mental health (*β* = − 0.00, 95% *CI* = [− 0.06, 0.02], *p* > .05), but we observed a significant interaction effect between altruism and perceived risk on mental health (*β* = 0.13, 95% *CI* = [0.05, 0.21], *p* < .001).

### Mediating effect of negative affect

We conducted an SEM to test our mediation hypothesis regarding whether the interaction effect between perceived risk and altruism on mental health was mediated by negative affect. Above, we determined that there was no interaction between perceived risk and positive affect; thus, we included negative affect as the only mediator in the model.

**Fig. 2:**
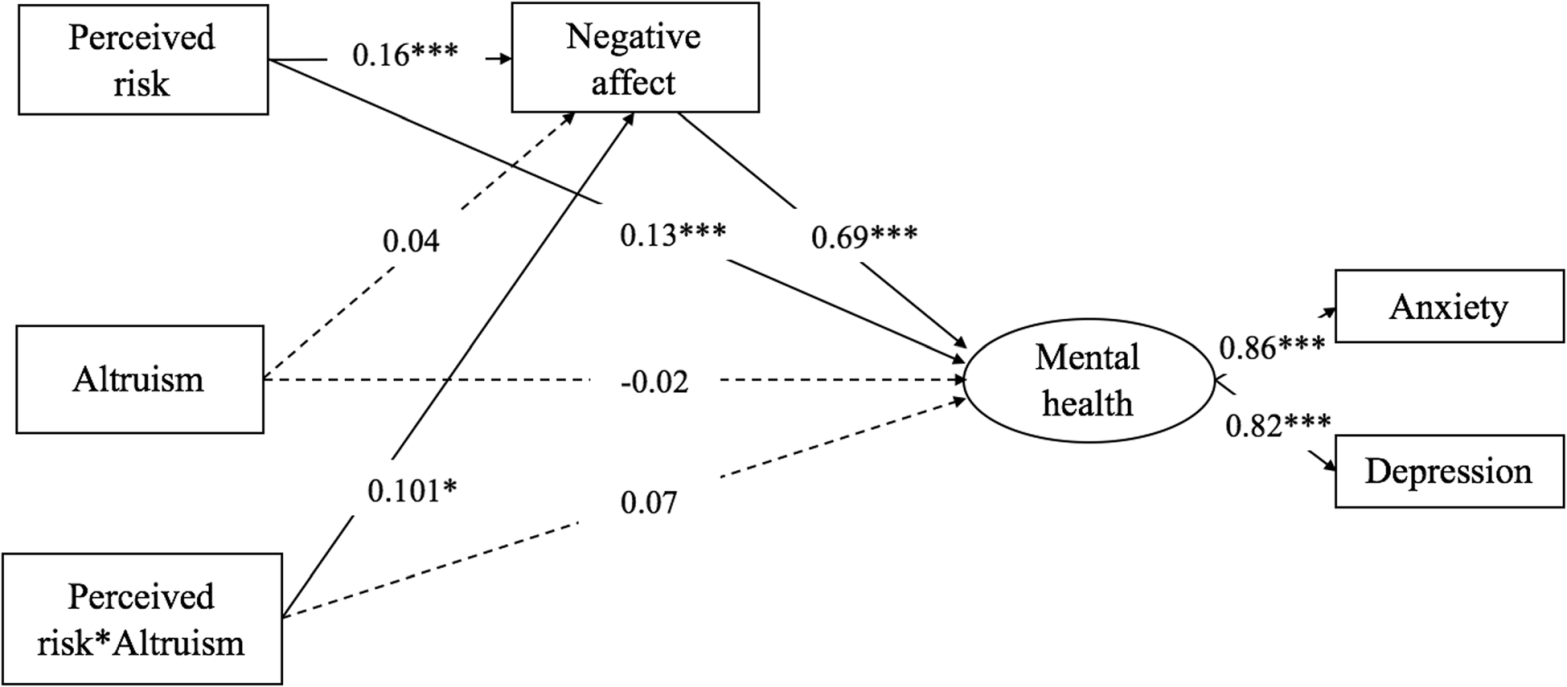
Final structural equation model. Note: The dashed lines represent the predictive paths that were non-significant. The solid lines represent a significant predictive effect. * indicates *p* < .05, ** indicates *p* < .01

Figure 2 shows the final SEM model, which fit the data well (*χ*^*2*^/*df* = 8.74, CFI = 0.963, TLI = 0.913, RMSEA (90% *CI*) = 0.076 [0.061–0.092], SRMR = 0.033). This model showed that a higher perceived risk predicted worse mental health via two paths as follows: one direct path (*β* = 0.13, 95% *CI* = [0.07, 0.21], *p* < .001) and one indirect path through negative affect (*β* = 0.16, 95% *CI* = [0.11, 0.21], *p* < .001). No direct effect of altruism on mental health was found (*β* = − 0.02, 95% *CI* = [− 0.06, 0.02], *p* > .05); thus, the interaction effect between altruism and perceived risk on mental health was entirely mediated by negative affect (*β* = 0.10, 95% *CI* = [0.03, 0.16], *p* < .05).

In summary, the moderating effect of altruism was completely mediated by negative affect, suggesting that when people perceive a high risk of COVID-19, those with high altruism may show worse mental health outcomes than those with low altruism because they may experience greater negative affect.

## Discussion

This study investigated the moderating role of altruism and the mediating role of negative affect on mental health during the outbreak of COVID-19 in China. Our results show that altruism is associated with increased anxiety and depressive symptoms, particularly when people perceive a higher risk of the virus. Negative affect provided an indirect path from altruism to mental health, suggesting that altruism plays a moderating role in worse mental health by increasing negative affect.

### Implications

The COVID-19 pandemic has presented a global health risk to humans. Most people perceive the threat of the virus in both frontline and low-risk areas, leading to generalized fear and fear-induced behaviour. In addition, people who perceive a high risk are more likely to show anxiety and depressive symptoms [33]. Our results verify the positive association between perceived risk and worse mental health. We provide empirical evidence for psychological interventions addressing risk perception in public health during the pandemic.

Altruism has been understood as an important protective mental health factor in previous studies [9, 34–36], while few studies have found a negative effect on mental health under specific circumstances. Our study presents a paradox in that altruism is associated with negative emotion and worse mental health outcomes when altruistic norms exist under conditions in which altruistic behaviour cannot occur. This result is consistent with the theory that altruism encompasses two core concepts, namely, attitude and behaviour [8]. There are two possible explanations for this phenomenon. On the one hand, the outbreak of COVID-19 was first identified in Wuhan, Hubei Province, China, and thousands of citizens were infected. The severity of this disease was much higher than that of SARS in 2003 or H1N1 in 2009. The Chinese government called for self-isolation and social distancing during the outbreak of the pandemic. Altruists who perceived a high risk of this pandemic and who could not help others experienced a dilemma, which might have reduced their self-efficacy and increased their feelings of helplessness and other negative emotions. On the other hand, altruists generally feel empathy; thus, if altruists perceive the risk to be very high, they might feel sorry and sad for those infected by the virus, which might increase their negative emotions. In summary, the strong benefits of altruism are based on the premise that altruistic behaviour exists.

The debate regarding the mechanism by which altruism impacts mental health, i.e., via the path of increased positive emotion, decreased negative emotion or both, remains controversial [18]. Our study explored the role of these paths by examining altruism as a moderator. There was a significant interaction effect between altruism and perceived risk on negative affect, whereas there was no interaction effect on positive affect. In addition, the effect of altruism and perceived risk on mental health was completely mediated by negative affect. This result suggests that the influence of altruism on mental health occurred entirely through the effect of altruism on negative affect. Although our study does not provide a direct explanation of the mechanism by which altruism results in better mental health, we provide evidence regarding the mechanism by which altruism leads to worse mental health by increasing negative emotions.

In addition to its theoretical implications, this study provides practical insight into public health management. First, we tested the influence of the social distancing policy on altruists. Due to the Chinese government’s social distancing policy, the university in this study strictly required each student to report his or her location to the school daily via a mobile phone to ensure that most university students self-isolated at home and to avoid mass gatherings. The results of this study offer some reflection regarding future regulations related to social isolation; more flexible regulations may be needed for altruists. For example, during the implementation of a social distancing policy, a psychological assistance hotline could be provided by the government to reduce the anxiety and depression caused by isolation. Second, altruistic behaviour can be performed in indirect ways, such as through online donations and online sharing of personal hygiene knowledge. Engaging in indirect altruistic activities may also relieve altruists’ anxiety and depressive symptoms. Third, effective psychological prevention strategies for the COVID-19 crisis can be developed to reduce risk perception, such as decreasing exposure to COVID-19-related news on social media, engaging in daily physical exercise, maintaining regular dietary and sleep habits, and having good communication with family members and friends.

### Limitations and future directions

Despite these findings, this study is limited in several aspects. First, we recruited only university students as participants, who may not be representative of the entire population of altruists. Meanwhile, the majority of the participants in the current survey were female and Han, which might have skewed the results. Second, the sample size was not sufficient and may not have provided sufficient power to detect some effects. The participants were mostly from low-risk areas in China rather than Wuhan, the city most affected by the COVID-19 pandemic. We assume that participants from Wuhan may have perceived more risk and that the mechanism of perceived risk on mental health would be more complicated among participants from this city. Future research could test and refine our model with participants from Wuhan. Third, we clarified that the mechanism by which altruism leads to worse mental health during the outbreak of COVID-19 relied on the path of increased negative affect, but we did not determine the mechanism by which altruism impacted negative affect during this specific period. We assume that empathy or self-efficacy may mediate the role of altruism in negative affect. Future research could focus on exploring this underlying mechanism to better understand the influence of altruism on mental health. Finally, our model was limited in its ability to reveal the causality of the variables of interest. Future research could strengthen the causal explanations by manipulating the variable of perceived risk in laboratory experiments.

## Conclusions

Our study focused on health psychology to understand how people responded to the threat of COVID-19, especially in terms of anxiety and depressive symptoms, which are widely considered to be indicators of negative mental health. Using representative data collected during the outbreak of COVID-19 in China, we took advantage of a unique opportunity to investigate the relationship between altruism and perceived risk in relation to emotion and mental health. The negative influence of the perceived risk of COVID-19 and altruism on mental health suggests that individual affect and mental health are greatly influenced by risk perception and that the protective effect of altruism requires specific conditions. In contrast to previous theory, out results suggest that altruism did not improve mental health during the epidemic but rather exacerbated affective symptoms, such as anxiety and depressive symptoms, which is an important contribution to previous theories of altruism. In addition, we examined the mechanism underlying the impact of altruism on mental health, further advancing theories of altruism. In summary, this study provides valuable insight into the psychological mechanism of altruism and a reference for public mental health during the pandemic. Future analyses could more deeply explore this mechanism and other factors that disturb people’s emotions and mental health.

## Data Availability

The datasets used during the current study are available from the corresponding author on reasonable request.
